# Detection of *Chlamydia psittaci* in the Genital Tract of Horses and in Environmental Samples: A Pilot Study in Sardinia

**DOI:** 10.3390/pathogens13030236

**Published:** 2024-03-07

**Authors:** Gaia Muroni, Elisa Serra, Giovanni Paolo Biggio, Daniela Sanna, Raffaele Cherchi, Andrea Taras, Simonetta Appino, Cipriano Foxi, Giovanna Masala, Federica Loi, Valentina Chisu

**Affiliations:** 1 Istituto Zooprofilattico Sperimentale della Sardegna, 07100 Sassari, Italy; gaia.muroni@izs-sardegna.it (G.M.); elisa.serra@izs-sardegna.it (E.S.); cipriano.foxi@izs-sardegna.it (C.F.); giovanna.masala@izs-sardegna.it (G.M.); 2 AGRIS, Servizio Ricerca Qualità e Valorizzazione delle Produzioni Equine, 07014 Ozieri, Italy; gbbiggio@agrisricerca.it (G.P.B.); danisanna@agrisricerca.it (D.S.); rcherchi@agrisricerca.it (R.C.);; 3 Dipartimento di Medicina Veterinaria, Università di Sassari, 07100 Sassari, Italy; simo@uniss.it; 4 Osservatorio Epidemiologico Veterinario Regionale della Sardegna, Istituto Zooprofilattico Sperimentale della Sardegna, 07100 Sassari, Italy

**Keywords:** *Chlamydia psittaci*, horses, zoonotic agents

## Abstract

The members of the *Chlamydiaceae* family are important pathogens that infect a wide range of vertebrate hosts, including humans. Among them, *Chlamydia psittaci*, historically considered as an avian agent, has recently been identified in livestock, primarily sheep and cattle, but also in horses, with the infection being linked to reproductive disorders, such as abortion, absorption of embryos, stillbirth, and the birth of weak foals. Much less is known about chlamydial infections in the Sardinian equine population. This study aimed to identify the chlamydial diversity in genital samples from asymptomatic Sardinian horses. However, some horses had a previous history of reproductive disorders, i.e., abortion and infertility. A total of 60 horses (39 mares and 21 stallions) were opportunistically recruited from 17 equine farms in central-northern Sardinia. Vaginal and uterine swabs from mares and urethral swabs and seminal fluid from stallions were sampled for the presence of chlamydial DNA. Samples from environments where the horses lived were also tested for the detection of *Chlamydia* spp. Eight vaginal swabs (8/39; 20%), two uterine swabs (2/27; 7%), two seminal fluid samples (2/20; 10%), and one urethral swab (1/21; 4.7%) were found to be positive for *Chlamydia* spp. by PCR analysis. In addition, results from environmental samples showed the presence of *Chlamydia* spp. in three environmental swabs (3/8; 37.5%) and five water samples (5/16; 31.2%). Sequencing results revealed that strains here identified were 99–100% similar to members belonging to the *Chlamydiaceae* family, including *C. abortus*, *C. psittaci*, and uncultured *Chlamydia* genotypes. *ompA* species-specific PCR performed on samples was found to be positive after 16S rRNA amplification gave positive results for *C. psittaci*. These results reveal the first presence of *C. psittaci* in the genital tract of horses and in the environment in Sardinia and indicate that this pathogen could be the prevailing cause of infertility and abortion in the tested equines. However, these findings need further proof and highlight the importance of adopting a ‘One Health’ approach to control the presence of this zoonotic bacteria in domestic animals in order to understand its impact on people exposed to the infection risk.

## 1. Introduction

The order Chlamydiales consists of obligate intracellular Gram-negative bacteria that infect an extensive range of domestic and wildlife hosts including birds, mammals, marsupials, amphibians, and reptiles [1,2,3]. To date, this order consists of nine families: *Chlamydiaceae*, *Clavichlamydiaceae*, *Cribchlamydiaceae*, *Parachlamydiaceae*, *Parilichlamydiaceae*, *Piscichlamydiaceae*, *Rhabdochlamydiaceae*, *Simkaniaceae*, and *Waddliaceae*. The *Chlamydiaceae* family currently consists of eleven *Chlamydia* species with variable pathogenicity and different preferential host [4]: *C. abortus*, *C. avium*, *C. caviae*, *C. felis*, *C. gallinacea*, *C. muridarum*, *C. pecorum*, *C. pneumoniae*, *C. psittaci*, *C. suis*, and *C. trachomatis*, as well as three species at *Candidatus (Ca.)* status: *Ca*. C. ibidis, *Ca*. C. sanzinia, and *Ca*. C. corallus. The newly described *Chlamydiifrater* genus comprises the species named *Chlamydiifrater phoenicopteri* sp. nov. and *Chlamydiifrater volucris* sp. nov. [4]. Among them, *C. trachomatis* and *C. pneumoniae* species are primarily human pathogens, while the other species are bacteria of veterinary interest that infect a variety of wild and domestic animals and that can be occasionally transmitted to human [5]. *Chlamydia psittaci* infection has been reported in several species of birds (which represent the main reservoir of the zoonotic pathogen) and in non-avian hosts [6]. In humans, the bacterium causes mainly respiratory infections with fever, chills, headache, myalgia, nonproductive cough, and respiratory distress as the most frequently reported clinical symptom [7]. Gastrointestinal symptoms and skin rashes have been also observed [7]. Rare complications could include myocarditis, encephalitis, hepatitis, keratoconjunctivitis, acute respiratory distress syndrome, and multiple-organ failure, with development of ocular lymphoma [8]. Serious or life-threatening illness can occur when the signs of psittacosis are not recognized [9,10]. The main route of human infection is by the inhalation of aerosolized organisms from dried avian feces or respiratory tract secretions of infected birds [11]. Other means of exposure include mouth-to-beak contact and handling of infected birds’ plumage and tissues. Rarely, human-to-human transmission has been reported with the epidemiology of human infections that always reflects the circulation of the bacterium in animal reservoirs [12]. Several studies have recently detected and performed molecular characterizations of *C. psittaci* in fetoplacental material in horses [13,14]. Also, the prevalence of *C. psittaci* has been evaluated in healthy mares/foals [15]. Horses are considered occasional hosts of *C. psittaci*, and the infection has been associated with pneumonia, conjunctivitis, and abortion [16]. In the literature, it is clearly stated that *C. psittaci* is a known cause of equine abortion and reproductive loss, and the association between *C. psittaci* and equine abortion has been evaluated with zoonotic implications for humans [2,17].

Moreover, although *C. psittaci* is an obligate intracellular bacterium, it has been documented in environmental samples and waters as reticulate bodies that may represent a long-term environmental reservoir of the bacterium [9]. Although previous studies in infected mares reported reproductive (such as genital infection, occasional abortion, and infertility) and respiratory signs (pneumonia, conjunctivitis, and polyarthritis) attributed to *C. psittaci* [18,19] and rarely to *C. abortus*, the relationships between equine abortion and chlamydial infection are still being studied [20]. This pilot epidemiological study investigated the presence of *Chlamydia* species in apparently healthy horses by also providing preliminary information about their potential role as reservoirs and sources of zoonotic infection. The results arising from this study would represent the evidence-based information necessary to carry out a large-scale study aimed at estimating the true prevalence of chlamydial infections in Sardinian breeding horses.

## 2. Materials and Methods

### 2.1. Study Area

Sardinia is an island of Italy with a total area of 24,100 km^2^, characterized by various eco-systems because of the presence of mountains, forests, plains, largely uninhabited areas, waterways, long sandy beaches, and rocky shores. Furthermore, the island is characterized by many autochthonous animal species. Among them, there are three horse breeds native to the island, named Giara, Sarcidano, and Sardinian Anglo-Arab, that are classified as “at risk” by the FAO (http://www.fao.org/dad-is accessed on 24 January 2024) if considering their local status. To date, 19.510 horses are regularly recorded in the Veterinary National Database and associated with 11.475 farms (mean = 2 horses/farm, SD = 0.9, min = 1, max = 50), with a density of 0.5 farms and about 0.7 horses per km^2^ [Sistema Informativo Veterinario-Statistiche. Patrimonio zootecnico equini, available at: https://www.vetinfo.it/j6_statistiche/#/report-pbi/33 (accessed on 24 January 2024)].

Considering the need to gather prior information about the prevalence of chlamydial infections in Sardinian breeding horses, a pilot study was planned and developed to evaluate possible critical steps in the process of intervention and sample testing. A small-scale test of the methods and procedures was carried out to evaluate the feasibility of a prevalence study, without claims of inference. On the basis of the results obtained, larger-scale tests could be performed in future studies. Thus, sample size calculation was not performed, and participation in this investigation was solely on a voluntary basis.

**Figure 1:**
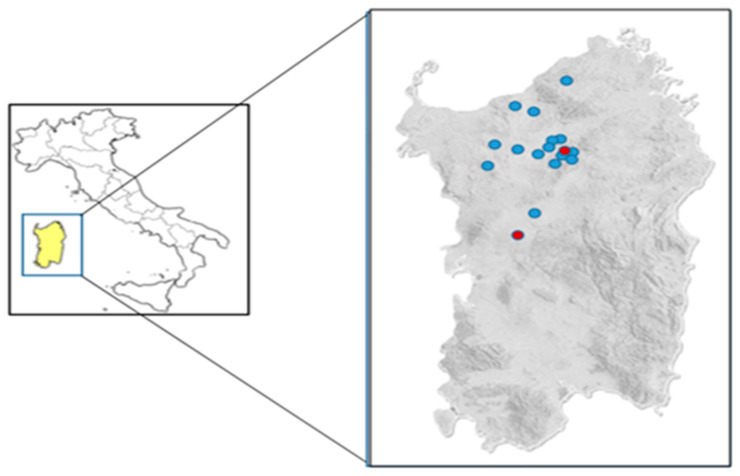
Map of the farms included in this study. Light blue dots: farms where samples were collected. Red dots: farms found to be positive for *Chlamydia* spp. after PCR analyses.

During the period February 2021 to May 2022, horse and environmental samples were collected from 17 horse centers located in the central-northern part of Sardinia as shown in Figure 1. These locations were chosen based on the willingness of the owners to participate in the sampling, which was therefore carried out for convenience. Clinical swab samples were collected by veterinarians according to good practice and with the agreement of owners.

### 2.2. Origin of the Samples

A total of sixty horses (39 mares and 21 stallions) used for breeding were voluntarily included in this study. Baseline characteristics of these animals are reported in Table 1. The 39 mares were aged between 5 and 25 years old (mean = 14.3, SD = 5.1), including 17 Anglo-Arabians, 14 Standardbreds, 3 Arabians, 3 Thoroughbreds, and 2 Italian Saddles. The 21 stallions (12 Anglo-Arabians, 4 Arabians, 3 Thoroughbreds, 1 Oldenburg, and 1 Luxembourg Warmblood) were aged between 6 and 29 years old (mean = 15.9, SD = 5.6).

Ethical review and approval were waived for this study, as this study did not involve any animal experiments. Samples were collected from horses using standard procedures as suggested by AGRIS, Servizio Ricerca Qualità e Valorizzazione delle Produzioni Equine, Ozieri, Italy, and submitted to the Experimental Zooprofilactic Institute of Sardinia for *Chlamydia* screening. Special authorization for sampling activities was not necessary; this action is regulated by the Italian Ministry of Health and performed in the case of infectious diseases.

### 2.3. Sampling Protocol

A qualified veterinarian conducted an accurate physical examination of each horse assessing overall body condition, skeletal development, state of nutrition and muscular tone, sensory state, mucous membranes, hydration status, lymph nodes, temperature, pulse, breath, and organic functions [21]. All horses from this study were also evaluated by ultrasonography of the reproductive tract, using a 5 MHz linear probe.

#### 2.3.1. Blood Collection

Blood samples were taken from the jugular vein using EDTA-containing vials and stored at 4 °C during their transportation to the laboratory. A basic hematological test was performed by analyzing the red blood cell (RBC) count, hemoglobin (HGB), hematocrit (HCT), mean corpuscular volume (MCV), mean corpuscular hemoglobin (MCH), MCH concentration (MCHC), red blood cell distribution width (RDW), platelet, and white blood cell (WBC) count. The concentrations of alkaline phosphatase, aspartate aminotransferase, gamma-glutamyl transpeptidase, creatine kinase, total protein concentration, albumin, total bilirubin, and triglycerides [22] were also evaluated by blood biochemical analysis.

#### 2.3.2. Collection of Swabs

During the estrous follicular phase, uterine (*n* = 27) and vaginal swabs (*n* = 39) were collected from each mare. Prior to the swab collection, an accurate disinfection of the vulva and perineal area was performed with povidone iodine (Betadine^®^, MEDA Pharma S.p.A., Milan, Italy) [23]. The urethral swabs from 21 stallions were also collected after cleaning of the penis with warm clean water to remove smegma, dead skin cells, and other debris. The penis was then dried using disposable paper [23].

The urethral swab was then inserted into the urethra to a depth of 2–4 cm and turned several times. Seminal fluid was also collected from the stallions using a “home-made” artificial vagina (INRA modified) with the stallion mounting a Franches-Montagnes mare or a phantom depending on the stallion [24]. The collected samples were stored at −20 °C until analyses.

Eight collection areas were selected as follows: the senior mares’ area, the mares’ pond, the foals’ area, the service center, the general pond, Garden 1, Garden 2, and the stallions’ area. For each area of collection, one environmental swab and two samples of water (either directly from the pond or from the drinkers) were obtained. Dry swabs were streaked in the selected area, collected at 4 °C in sterile wrapping; around 1.5 mL was held in a microtube (Eppendorf, Hilden, Germany) at 4 °C and then transferred to the IZS laboratory for further examination.

#### 2.3.3. Ectoparasite Collection

As part of the control ticks and tick-borne diseases program, ectoparasites were also collected from the sampled horses following the removal guidelines of the Centers for Disease Control (CDC; https://www.cdc.gov/, accessed on 24 January 2024). Fine-tipped tweezers were used, and ticks and flies were placed in vials at room temperature. All samples were transported to the laboratory of Istituto Zooprofilattico Sperimentale della Sardegna (IZS) in a refrigerator at a temperature of 4 °C within 4 h of sample collection and stored at −20 °C, until identification. An expert in entomology and acarology, using the taxonomic keys by Estrada-Peña et al. [25], carried out a morphological identification using a binocular microscope (10–50×).

### 2.4. Direct Immunofluorescence Test

To determine the presence of *Chlamydia*, vaginal, uterine, urethral, and environmental swabs were analyzed by direct immunofluorescence (IFD) using a commercial kit (IFD; IMAGEN *Chlamydia* test; Oxoid Ltd., Basinstoke, UK). Before analysis, the swabs were rolled, by pressure, on the microscope slide. The slides were examined and interpreted using an epifluorescence microscope. The results were categorized as positive if extracellular elementary bodies (EBs) were visible as very small bright apple-green-fluorescent smooth-edged disc shapes approximately 300 nm in diameter. The background observed is usually made of red counterstained cells and cellular debris. A positive control slide, provided by the kit IMAGEN, was included in each IFD test.

### 2.5. DNA Extraction

DNA from swabs, seminal fluid, water samples, ticks, and flies was extracted using the commercial DNeasy Blood and Tissue Kit (QIAGEN, Hilden, Germany) following the manufacturer’s instructions with some modifications such as lysis in 600 μL ATL buffer and 60 μL Proteinase K for ectoparasites. The DNA extracted was stored at 4 °C until use.

### 2.6. Screening of Chlamydia spp. by PCR Amplification

All DNA samples were tested by three different PCR protocols. DNA of *Chlamydia abortus* (isolated from abortion product in Sardinia [26]) and *Chlamydia psittaci* (DNA provided by Centro di Referenza Nazionale Clamidiosi, IZS della Lombardia e dell’Emilia Romagna, Sezione Diagnostica di Pavia, Italy) and autoclaved water were used as positive and negative control, respectively, and included in each amplification protocol. The first assay allowed us to identify members of the *Chlamydiales* order by amplifying a 256-bp fragment of the 16S rRNA gene. The protocol was carried out using the primers 16SFor2 (5′ CGTGGATGAGGCATGCAAGTCGA 3′) and 16SRev6 (5′ ATCTCTCAATCCGCCTAGACGTCA 3′) as previously described [27]. The amplification was performed in an automated DNA thermal cycle (GeneAmp PCR Systems 2400 and 9700; Applied Biosystems, Courtaboeuf, France) with a temperature profile as follows: an initial denaturation at 95 °C for 15 min, followed by 40 cycles of denaturation at 94 °C for 60 s, annealing at 60 °C for 30 s, and extension at 72 °C for 60 s, with a final extension step at 72 °C for 5 min. PCR products were verified by electrophoresis in 1.5% agarose gel stained with SYBR™ Safe DNA Gel Stain (Thermo Fisher Scientific Inc., Waltham, MA, USA) and then watched under UV transillumination in an Image Master VDS-CL System (Amersham Biosciences Europe GmbH, Milano, Italy).

The 16S rRNA positive DNA samples were successively submitted to the second PCR protocol described by Chisu et al. [27], which allows us to identify *C. abortus* using oligonucleotide primers pmp1 (5′-ATGAAACATCCAGTCTACTGG-3’) and pmp2 (5′-TTGTGTAGTAATATTATCAAA-3′), which amplify a 318 bp fragment of the *pmp* gene. Each reaction consisted of 12.5 µL of QuantiTect Probe PCR Master Mix (Qiagen, Toronto, ON, Canada; 1X final concentration), Milli-Q water RNAse-free (9.5 µL), 1 µL of each forward and reverse primer (25 pmol/µL), and 1 µL of DNA template, in a final volume of 25 µL.

The 16S rRNA positive samples were also submitted to the third PCR protocol that employed the oligonucleotide primers Cpsitt F (5′-GCTACGGGTTCCGCTCT-3′) and Cpsitt R (5′-TTTGTTGATYTGAATCGAAGC-3′), which amplified a 1041 bp fragment of the *omp*A gene specific to *C. psittaci* species [28].

### 2.7. Purification and Sequencing

The 16S rRNA and *omp*A amplified products were purified using the QIAquick Purification Kit (QIAGEN, Hilden, Germany) according to the manufacturer’s protocol. The purified amplicons were bidirectionally sequenced using an ABI Prism BigDye Terminator Cycle Sequencing Ready Reaction Kit (Applied Biosystems, Foster City, CA, USA). The obtained forward and reverse reads were corrected using ChromasPro software version 2.2 (Technelysium, Helensvale, Australia). Consensus sequences were aligned with CLUSTALX [29] and then compared with similar sequences available in the GenBank database using the BLASTn (Nucleotide BLAST) search tool (http://blast.ncbi.nlm.nih.gov/).

The generated *omp*A sequences were then deposited in Gen Bank under the accession number PP429546-PP429550.

### 2.8. Statistical Analysis

Considering the non-inferential purpose of this work, descriptive statistics of the samples collected and the associated laboratory results are presented. The analysis was stratified between sex (mares or stallions) and young (1–6 years of age), middle (7–14 years of age), and older horses (more than 15 years of age). Descriptive statistics were calculated providing mean and standard deviation (SD) or medians with interquartile ranges for continuous variables, frequency, and percentage for categorical variables.

With the main aim of providing valid information essential for sample size calculation of future research, any associations between baseline features and laboratory test results were investigated. Either the Chi-square test or the Fisher exact test was applied to compare qualitative variables. The Kruskal–Wallis nonparametric test was applied to compare differences between quantitative variables.

The agreement between the methods applied to collect DNA from mares (vaginal and uterine swabs) and stallions (urethral swab and seminal fluid) was first evaluated in a contingency table. The degree of accuracy and reliability in positive/negative cases classification was evaluated applying the Cohen’s kappa coefficient [30], and the 95% CIs were calculated by the method proposed by Fleiss et al. [30]. The kappa coefficients were evaluated using the guideline outlined by Landisand Koch [31], where the strength of the kappa coefficients is slight if K = 0.01–0.20, fair if K = 0.21–0.40, moderate if K = 0.41–0.60, substantial if K = 0.61–0.80, and almost perfect if K = 0.81–1.00.

The preliminary results of this study were used to calculate the sample size of horses to be included in a population-based study aimed at estimating the true prevalence of *Chlamydia* spp. in Sardinia, with a specified level of confidence and precision, assuming a test with imperfect sensitivity and specificity. First, true prevalence was estimated using the prevalence found in this study as assumed. Inputs were the sample size, the number of positives, the desired level of confidence (95%), the desired precision of the estimate (0.05%), and the assumed values for sensitivity (0.7) and specificity (0.9) of the test. Confidence limits for apparent and true prevalences were calculated [32,33]. Subsequently, the population-based study sample size was calculated for an assumed large (infinite) population.

All statistical analyses were performed using R software (Version 4.1.2, R-Foundation for Statistical Computing, Vienna, Austria). Sample size calculation was solved by using the *epiR* package of R software (https://CRAN.R-project.org/package=epiR, accessed on 24 January 2024). A two-tailed *p* value < 0.05 was considered statistically significant.

Based on these preliminary results, a hypothetical sample size able to estimate the prevalence of *Chlamydia* in the whole of Sardinia was calculated.

## 3. Results

### 3.1. Origin of the Samples and Detection of Chlamydia Species by Molecular and Serological Tests

After thorough clinical examinations and blood analyses, no abnormalities in the general physical status, hematological and biochemical profiles, or cultural and serological test were observed. The mares did not show any ultrasonographic abnormalities.

All uterine, vaginal, urethral, and environmental swabs were found to be negative in the direct immunofluorescence test.

Out of all the analyzed horses, 13 (13/60; 21.6%) tested positive for chlamydial DNA by using the 16S rRNA fragment gene (Table 2). Specifically, the highest number of positive results were recorded from vaginal swabs (8/39; 20.5%), followed by seminal fluid (2/20; 10%) and uterine swabs (2/27; 7.4%). Moreover, one sample from a urethral swab (1/21; 4.8%) and two from seminal fluids (2/16; 10%) tested positive for *Chlamydia* spp. after molecular analyses (Table 2). Sanger sequencing of the 16S rRNA amplicons generated clear electropherograms for all sequences that exhibited 100% identity with uncultured *Chlamydia* sp. (GenBank accessions: MT581449), *Chlamydia abortus* (GenBank accessions: CP024084), and *Chlamydia psittaci* (GenBank accessions: CP025423) isolated from various hosts worldwide.

Post-amplification and Sanger sequencing of the *omp*A fragment gene generated 11 sequences that showed 100% identity with *omp*A *C. psittaci* strains isolated from ducks in China (Genbank accession number MK630234). No positive results were highlighted for *C. abortus* (Table 2).

No statistically significant differences were detected between mares and stallions, between age groups, or between breed groups in the probability of being PCR-positive (*p*-values > 0.05). However, out of the 10 mares that were found to be positive for *C. psittaci* in this study, nine showed at least one fertility problem. From the preceding season, six barren mares, one fetal resorption, and two cases of endometritis were recorded.

Concordance between PCR tests for chlamydial DNA was evaluated, and the results are reported in Table 3: (a) for mares and (b) for stallions.

As reported in Table 3a, the results of the PCR tests performed by vaginal and uterine swabs in mares highlight the higher ability of vaginal swabs to identify positive samples (four positive samples, of which three were negative in uterine swabs) and good agreement between the tests in identifying negatives (22 samples identified as negatives), with an observed agreement of 85%. The Cohen’s K coefficient of 0.25 indicates a fair agreement between tests. In stallions (Table 3b), a substantial agreement of 87.5% was found by comparison between urethral swabs and seminal fluids, with moderate agreement between tests (Cohen’s K coefficient = 0.45).

### 3.2. Detection of Chlamydia Species in the Environment

The 16S rRNA PCR was applied to 24 DNA sequences extracted from environment swabs and water samples. Specifically, three swabs collected from the general pond, the service center, and Garden 1 were found to be positive for *Chlamydia* spp. (3/8; 37.5%). Chlamydial DNA was also detected in five water samples (5/16; 31.5%) collected from the senior mares’ area, the mares’ pond, the stallions’ area, and Garden 2 (*n* = 2). The results are summarized in Table 4.

We succeeded in sequencing the 16S rRNA gene sequences from 8 to 24 positive samples that matched uncultured *Chlamydia* sp. (GenBank accessions: MT581449), *Chlamydia abortus* (GenBank accessions: CP024084), and *Chlamydia psittaci* (GenBank accessions: CP025423) isolated from various hosts worldwide in BLASTN searches. However, sequencing was not achieved for four environmental samples that generated an unclear chromatogram and were excluded from the analysis. Due to the high degree of similarity with the strains as above, *C. abortus* and *C. psittaci omp*A- and *pmp*-specific PCR was applied to all positive samples. The *omp*A genotyping was then applied to eight *C. psittaci*-positive samples. As a result, the nucleotide sequence identities of the *omp*A gene between the isolates was 100% identical to *Chlamydia psittaci* strains (GenBank Accession Number: MK630234) as shown in Table 4.

All of the ectoparasites examined in this study were removed from mares and were morphologically identified as *Rhipicephalus* spp. (*n* = 1), *Hyalomma marginatum* (*n* = 26), *Dermacentor marginatus* (*n* = 1), *Rhipicephalus sanguineus* (*n =* 19), and *Hippobosca equina* (*n* = 5). There were no detectable ticks on stallions at the time of sample collection.

All the ticks and horse flies turned out negative after PCR tests (Table 5).

### 3.3. Sardinian Survey Sample Size Calculation

Using a sample size of 60 horses, of which 13 were positive for *Chlamydia* spp., 95% of the desired confidence level and a desired precision of the estimate of 0.05%, and assuming a test sensitivity of 0.7 and specificity of 0.9, the apparent prevalence of *Chlamydia* spp. was equal to 21.7% (95% CI = 13.1–33.6), while the true estimate was 25.6% (95% CI = 12.5–44.0).

A one-stage cluster-sampling approach was applied to estimate the prevalence of *Chlamydia* spp. considering a test with imperfect sensitivity and specificity, considering the Sardinian horse farms as a cluster. Sampling stratifications were not included given that all comparisons between sex, age, and breeds did not produce statistically significant results in the probability of detecting *Chlamydia* spp.

**Figure 2:**
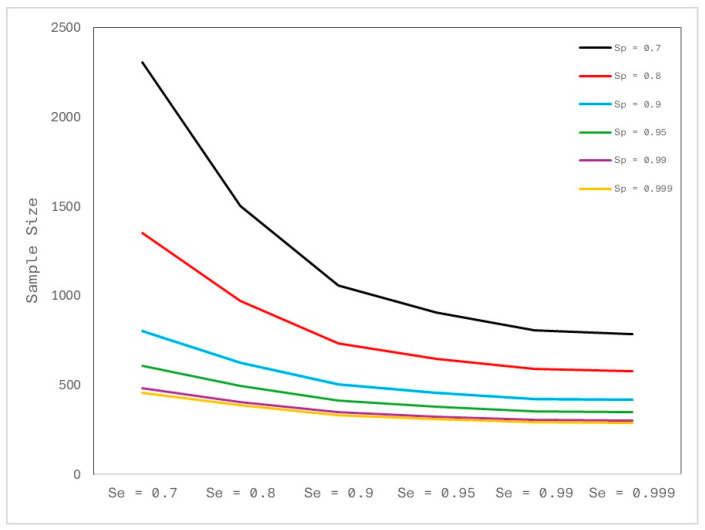
Samples sizes required for true prevalence = 0.25, precision = 0.05 and a range of values of sensitivity and specificity.

Figure 2 illustrates the sample size required to estimate the true prevalence with a specified level of confidence (95%) and precision (0.05%), considering an overall number of 11.475 horse farms in Sardinia and hypothesizing an assumed true prevalence of 25.6% (95% CI = 12.5–44.0). Supposing to apply a test with a sensitivity of 0.7 and a specificity of 0.9, about 800 farms should be included and sampled to estimate the true prevalence of *Chlamydia* spp.

## 4. Discussion

Recent advances in understanding the etiopathogenesis of *Chlamydia psittaci* in nature have provided a new perspective on the biology and evolution of this microorganism in the equine species [34]. Although primarily a pathogen of birds, from which infection can spill over into humans and other mammalian hosts, *C. psittaci* has been also identified as an important cause of reproductive loss in equines [35,36,37]. The disease results in late-term abortions and neonatal illness, and zoonotic pathogens can spread to humans through any contact with *C. psittaci*-infected horses or tissues [35]. However, there are several key questions concerning pathogen–horse interaction, as well as the management of genital infections and, above all, the presence of this microorganism in the environment. Currently, urogenital screening for *Chlamydia* spp. infection is generally performed only in cases of clinically apparent issues, thus neglecting the subtle role that various animal species play in this pathology.

The results of this pilot study revealed that prevalence of *C. psittaci* in vaginal swabs was significantly higher compared to uterine swabs. These results are consistent with previous studies [38,39], in which it was confirmed that vaginal swabs are the more appropriate specimens for diagnosis of genital tract infection with *Chlamydia* species in horses since this technique allows a greater quantity of mucus and cells to be collected and is less dependent on the operator’s skill [38].

The prevalence of *C. psittaci* in equine vaginal tracts has been previously reported [15]. Experimental infection performed on pregnant sheep with *C. psittaci* resulted in uteroplacental infections, but it also caused late-term abortion or birth to weak, low-birth-weight lambs. Moreover, infected ewes excreted chlamydial antigen during estrus, and the shedding persisted for over 1 year in vagina, uterus, and oviduct samples with endometrial cells in the basal stroma as the predominant site of infection [40]. All horses screened in this study did not show any classical signs of chlamydiosis despite several being *C. psittaci*-positive at genital sites. This was in accordance with recent studies where it was highlighted that asymptomatic infections are the most common, and *C. psittaci* infections in horses can result in reproductive disease which, if left untreated, may progress to infertility and abortion [35]. However, most of the positive horses here screened have a past medical history of reproductive disorders. Six of them failed to become pregnant and showed embryonic resorption as a clinical manifestation of reproductive disorders in one case. Endometritis was also diagnosed in two mares, and in one case, it was associated with edema. *Chlamydia psittaci* in infected mares has been described in a retrospective study where the pathogen was detected in association with equine abortion cases in Australia [17]. We cannot evaluate if *C. psittaci* is an emerging cause of equine infectious abortion or an underdiagnosed infection in Sardinia. Future retrospective studies would confirm this point as well as the involvement of *C. psittaci* in equine reproductive disorders. The greater positivity observed in the seminal vesicles is probably due to a greater presence of the microorganism in these tissues, which act as reservoirs in urogenital transmission [41].

An abundance of *Chlamydia* species belonging to the *Chlamydiaceae* family and including *C. psittaci* has recently been identified in Sardinian tick species [27], suggesting they may be a vector of infection. However, ticks removed from horses and tested here by a validated chlamydiales-specific PCR gave negative results for *Chlamydia* species. The presence of *C. psittaci* in ticks from horses should be further explored considering their vector potential.

The molecular results also confirmed the circulation of *C. psittaci* strains in environmental samples, specifically in water. Since the circulation of this species has not previously been reported in the Sardinian environment, this result represents the more interesting finding in this study.

The *omp*A sequences generated in this study shared the same genotypes as those in the SZ15 *C. psittaci* strain from Asian ducks [42]. The same strain has been recently described in humans with pneumonia in Zhejiang Province [43], and these findings suggested that *C. psittaci* may infect humans in different regions via bird migration. Moreover, generated *omp*A sequences also showed 99% similarity to *omp*A from ST24 (6BC) strains, a well-described avian *Chlamydia* type strain of considerable public health importance [44,45]. This is the dominant strain typed to date in Australian equine samples, psittacine birds, and humans by multilocus sequence typing (MLST) that emerged as the “gold standard” methodology for molecular characterization of strains [46,47].

Importantly, the *C. psittaci* strains here obtained from the environment were the same as those detected in the horses here screened. In this context, an indirect transmission from a contaminated environment to *C. psittaci*-infected horses presumably via fecal contamination of non-migratory avifauna could be hypothesized. In fact, although *C. psittaci* needs a host during the replicative phase, its elementary bodies can persist in soil and water following shedding from infected birds [48]. However, this remains speculative, since no samples from wild birds have been included in this study. These results emphasize the need to adopt surveillance measures to prevent the spread of *C. psittaci* infection between horses and to decrease the risk of human infection. This information has been partially reported by other authors; however, they did not report the traceability of the strain in both the environmental samples and the examined subjects [49,50].

In addition, the identical sequences of *C. psittaci* recently reported in parrots housed in a pet shop in South Sardinia [51] indicate the spread of this strain in the island and the need of planning more control strategies and prevention in the study area. Human disease caused by *C. psittaci* is often under-reported, and complete information about the patients is not always available.

As previously observed in the literature, the results obtained through direct immunofluorescence have demonstrated that nucleic-acid-based detection methods are more sensitive and can detect low levels of this microorganism [36]. This indicates that there are differences in the sensitivity and specificity of different diagnostic tools used to reveal chlamydial infections in horses. However, some diagnostic tools could not detect the presence of *C. psittaci*, which leads to the misidentification of this pathogen including the underestimation of the prevalence of chlamydial infections. Therefore, the negative IFD results may reflect the fact that these matrices are not the best target for the detection of *Chlamydia* species in equids. The positive results obtained with DNA-based methods confirm the test’s sensitivity differences. On the other hand, a positive serological test indicates the exposure to these microorganisms rather than actual evidence of bacterial replication.

Although the *C. psittaci omp*A genotyping method is limited in its sensitivity in comparison with qPCR assays (which are the current diagnostic standard in the field), we rely on this limitation to provide preliminary characterization of *C. psittaci* strains circulating in Sardinia. Considering the strong relevance of psittacosis for human health, the diagnosis of *C*. *psittaci* by using Cps-specific qPCR assays will be necessary to provide more sensitive detection and greater specificity of the pathogen.

Moreover, the negative *pmp* test results for the detection of *C. abortus* emphasize the hypothesis of the involvement of other chlamydial species than *C. psittaci* and *C. abortus* in Sardinia.

To the best of the authors’ knowledge, this is the first study in Sardinia in which *C. psittaci* was detected in the genital tracts of horses and in environmental samples. Evaluating mares and stallions for the presence of *C. psittaci* (by laboratory examination of semen or other samples from the reproductive tract) prior to each breeding season is a highly recommended practice to avoid an asymptomatic carrier state in the stallion. Considering that the clinical manifestation of *C. psittaci* in horses is extremely rare, we will continue with this line of research to confirm the implication of this pathogen in the reproductive disorders of equids.

## 5. Conclusions

These results indicate the first evidence of *C. psittaci* in mares, stallions, and environmental samples. Due to the recognized zoonotic potential of this microorganism and to the still-unclear aspects of the pathology in horses, it is necessary to deepen the epidemiology of this disease and increase its biosecurity aspects. It would be appropriate to pay more attention to the presence of this microorganism in equine herds through more assiduous monitoring, including *C. psittaci* differential diagnosis and the routine application of the vaginal test in females and males.

## Figures and Tables

**Table 1:** Baseline features of the horses involved in the study, by mares (*n* = 39) and stallions (*n* = 21). Data are presented as a number (percentage).

Features	Mares (*n* = 39)	Stallion (*n* = 21)
Breeds		
Anglo-Arabian	17	12
Standardbred	14	0
Arabian	3	4
Thoroughbred	3	3
Italian Saddle	2	0
Oldenburg	0	1
Luxembourg Warmblood	0	1
Age		
Young (1–6 y)	1	1
Middle (7–14 y)	19	10
Old (>15 y)	19	10

**Table 2:** Results of PCR for *Chlamydia* spp., *C. abortus*, and *C. psittaci* performed on vaginal, uterine, and urethral swabs and seminal fluid collected from the 39 mares and 21 stallions involved in this study. Data are presented as a number (percentage), calculated by sample type.

Gender	Sample Type	Method	*Chlamydia* spp. (*16S rRNA* Screening)	*C. abortus* (*pmp* Assay)	*C. psittaci* (*ompA* Assay)
Mares (39)	Vaginal swabs (39)	PCR-positive samples	8 (20.5%)	0	8 (20.5%)
	Uterine swabs (27)	PCR-positive samples	2 (7.4%)	0	2 (7.4%)
Total in mares			10 (25.6%)	0	10 (25.6%)
Stallion (21)	Urethral swabs (21)	PCR-positive samples	1 (4.8%)	0	1 (4.8%)
	Seminal fluid (16)	PCR-positive samples	2 (10%)	0	0
Total in stallions			3 (14.2%)	0	1 (4.8%)
Overall results			13 (21.6%)	0	11 (18.3%)

**Table 3:** Agreement table matrix. (a) Contingency table between PCR tests performed on vaginal swabs and uterine swabs in mares; (b) contingency table between PCR tests performed on urethral swabs and seminal fluids in stallions. Agreement values are presented as overall agreement frequency, Cohen’s kappa coefficients, and 95% confidence intervals (95% CI).

a	Uterine Swabs			
**Vaginal Swabs**	**POS**	**NEG**	**Total**	**Substantial Agreement: 85%**
POS	1	3	4	Cohen’s k: 0.25 [95% CI = 0.13–0.44]
NEG	1	22	23	
Total	2	25	27	
**b**	**Seminal Fluid**			
**Urethral Swabs**	**POS**	**NEG**	**Total**	**Substantial Agreement: 87.5%**
POS	1	0	1	Cohen’s k: 0.45 [95% CI = 0.37–0.52]
NEG	2	13	15	
Total	2	14	16	

**Table 4:** Environmental samples collected in the farm involved in this study.

Environmental Samples (*n*)	Collection Areas	IFD *Chlamydia* spp.	16S rRNA PCR *Chlamydia* spp.	*pmp* PCR *C. abortus*	*omp*A PCR *C. psittaci*	GenBank Accession Number (1st Hit; ID%)
Swabs (8)	Senior mares’ area Mares’ pond Foals’ area Service center General pond Garden 1 Garden 2 Stallions’ area	0/8	3/8	0/8	1/8	*C. psittaci* (MK630234; 100%)
Water (16)	Senior mares’ area Mares’ pond Foals’ area Service center General pond Garden 1 Garden 2 Stallions’ area	0/16	5/16	0/16	3/16	*C. psittaci* (MK630234; 100%)

**Table 5:** Tick and fly identification. F = female; M = male; N = nymph; E = engorged; NE: not engorged.

Sample (*n*)	Species (*n*)	Tick Stage, Sex, and Engorgement State (*n*)	Host (*n*)	PCR *Chlamydia* spp.
Ticks (46)	*Rhipicephalus* spp. (1)	FE (1)	1	0
	*Hy. marginatum* (26)	FE (8); FNE (1); MNE (14); ME (2);	10	0
	*D. marginatus* (1)	MNE (1)	1	0
	*Rh. sanguineus* (19)	FE (7); FNE (1); MNE (5); ME (1); NE (3); NNE (2);	7	0
Flies (5)	*H. equina* (5)	F (5)	1	0

## Data Availability

Data are contained within the article.
